# THE SYNERGISTIC EFFECT OF STRESSORS AND UPLIFTS FOR DAILY SUBJECTIVE AGING IN THE US

**DOI:** 10.1093/geroni/igad104.1534

**Published:** 2023-12-21

**Authors:** Shevaun Neupert

**Affiliations:** North Carolina State University, Raleigh, North Carolina, United States

## Abstract

Exposure to daily stressors is consistently associated with reduced health and well-being, including feeling subjectively older. Experiencing uplifts, however, are typically associated with increased health and well-being, but have not yet been examined with respect to subjective aging. The current study tested daily stressors and uplifts as unique as well as interactive effects for daily subjective age and daily ageist attitudes. A sample of 440 U.S. adults aged 50-85 participated in a 14-day daily diary study, reporting on their subjective age, ageist attitudes, daily stressors, and daily uplifts each day. All variables demonstrated significant within-person variability, with ICCs ranging from .68 (uplifts) to .75 (subjective age). Daily stressors and daily uplifts were each uniquely predictive of daily ageist attitudes, where daily stressors were positively associated and daily uplifts were negatively associated with daily ageist attitudes. For daily subjective age, there was a synergistic effect of daily stressors and daily uplifts. On days with no stressors, daily uplifts were not associated with daily subjective age. However, on days with stressors, increases in daily uplifts were associated with significant decreases in subjective age. These findings underscore the multidimensionality of subjective age constructs and will be situated within cross-cultural comparisons.

